# Selective cannabis strain allergy in a patient presenting with a local allergic reaction

**DOI:** 10.1186/s13223-021-00552-3

**Published:** 2021-05-17

**Authors:** Peter Stepaniuk, Amin Kanani

**Affiliations:** Division of Allergy and Immunology, Department of Medicine, University of British Columbia, 905-750 Broadway W, Vancouver, BC V5Z 1H8 Canada

**Keywords:** Cannabis, Marijuana, Strain, Allergy, Indica, Sativa, Hybrid, Phylogeny, Taxonomy

## Abstract

**Background:**

Cannabis use is growing domestically due to recent legalization in many jurisdictions. There are two main species of cannabis, *Cannabis sativa* and *Cannabis indica*, and thousands of different commercially available cannabis strains. Although there are multiple reports of cannabis allergy in the literature, to our knowledge, there is no prior published report of selective cannabis strain allergy.

**Case presentation:**

A 31-year-old male was referred for allergy assessment due to several episodes of localized pruritus and erythema after direct contact with various strains of cannabis. He had noted that the severity of his reaction appeared to be strain dependent. He developed a severe local reaction involving bilateral periorbital edema shortly after coming into direct contact with one particular strain of cannabis. He denied any adverse symptoms after inhalation of cannabis. Fresh skin prick testing was performed to various strains of cannabis and had positive testing to the three of the five tested strains.

**Conclusions:**

We believe this is the first reported case of selective cannabis strain allergy based on patient history and skin prick testing. This case report outlines the variability in different strains of cannabis and stresses the importance of further research into cannabis allergen identification. Multiple cannabis allergens should be included and incorporated into commercial extracts when they become routinely available.

## Background

**Fig. 1:**
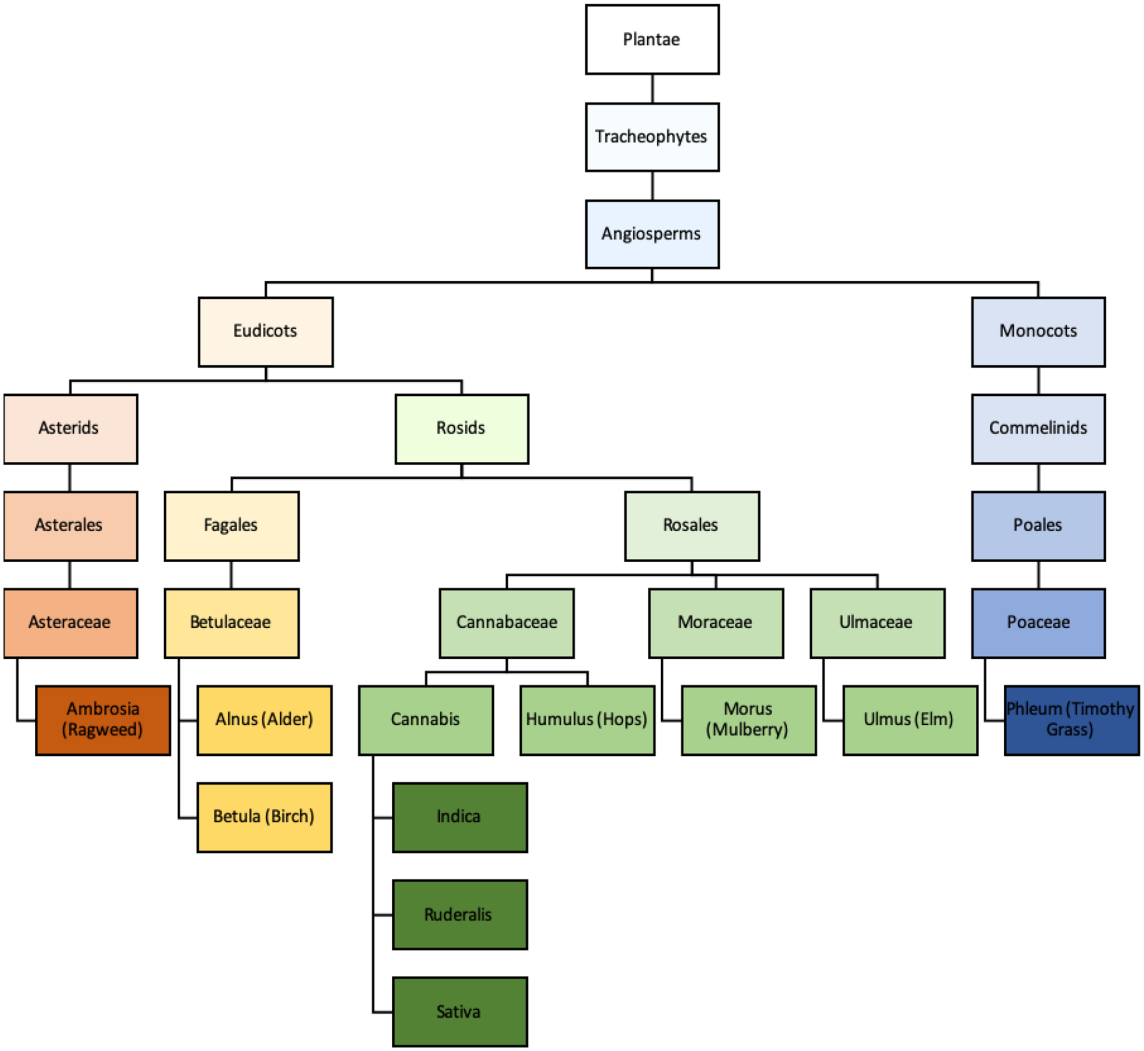
Taxonomy of cannabis relative to other common allergenic plants adapted from Canadian Society of Allergy and Immunology allergen immunotherapy manual [3–5]]

Cannabis use is prevalent across the world. In response to recent legalization in many jurisdictions, more information regarding potential adverse events are becoming evident. The three main species of cannabis are *Cannabis sativa, Cannabis indica* and *Cannabis ruderalis*. *C. indica* and *C. sativa* are more commonly used for their medicinal effects, and it is believed that all current commercially available strains are descendants of these two species [1]. New cannabis strains are produced by crossbreeding existing strains to take advantage of particular drug effects and a hybrid strain is considered to contain components of both *C. indica* and *C. sativa.* There are nearly 3000 different strains listed on some commercial websites which are organized into three dominant classes (Indica, Sativa, Hybrid) [2]. The cannabis plants are part of the family *Cannabaceae* and order *Rosales*, and are phylogenetically more closely related to common allergenic trees (specifically Hops, Mulberry and Elm), as opposed to other common allergenic plants (e.g., grasses and weeds) [3, 4] (Fig. 1). Various studies have linked cannabis use to hypersensitivity reactions including exacerbations of asthma, allergic rhinitis, contact dermatitis, anaphylaxis and urticaria [6–9]. However, on review of the literature, there have not been any reports of selective cannabis strain allergy that we could identify.

## Case presentation

A 31-year-old male was referred to our community allergy practice for assessment of suspected cannabis allergy. He had previously experienced several episodes of localized pruritus and erythema after direct contact with various strains of cannabis and noted that the severity of his reaction appeared to be strain dependent. He had developed a severe local reaction involving bilateral periorbital edema shortly after coming into direct contact with one particular strain of cannabis (believed to be Blue Moonshine, but patient uncertain). This particular episode was also associated with dyspnea in the absence of any other systemic symptoms. For this reaction he presented to a local emergency department and was treated with antihistamines and corticosteroids with good effect. He had never had an immediate reaction after smoking cannabis. After this particular episode and prior to his allergy consultation, he continued to smoke cannabis without adverse reaction but tried to avoid direct contact, particularly with his mucous membranes. His past medical history was notable for asthma and seasonal rhinoconjunctivitis.

The patient was diagnosed with recurrent local allergic reactions after direct contact with cannabis and he had a suspected selective cannabis strain allergy based on his clinical history. Skin testing was performed to environmental inhalants and the patient had positive reactions to dust mite, grass pollens and various tree pollens. The patient also brought in multiple strains of cannabis for skin testing including “Blue Moonshine” (*Cannabis indica* dominant strain), “Blue Dream” (Hybrid strain that is largely *Cannabis sativa* dominant), “Sweet Island Skunk” (*Cannabis sativa* dominant strain), “Sweet Skunk” (Hybrid) and “Blueberry Haze” (Hybrid). The cannabis strains where mixed with small aliquots of water for skin testing. Fresh testing was required due to the absence of commercially available abstracts. He had positive testing to the strains “Blue Dream” (7 mm), “Sweet Island Skunk” (10 mm) and “Blueberry Haze” (6 mm) while he had negative testing to “Blue Moonshine” and “Sweet Skunk” (Table 1). Histamine and saline controls were appropriate (3 mm and 0 mm respectively).

**Table 1:** Results of patient skin testing to select cannabis strains

Cannabis strain	Skin prick test size of wheal
Blueberry haze (H)	6 mm
Blue dream (H/S)	7 mm
Blue moonshine (I)	0 mm
Sweet island skunk (S)	10 mm
Sweet skunk (H)	0 mm

*H* hybrid, *S* *C. sativa* dominant strain, *I* *C. indica* dominant strain

The patient was advised to avoid direct cutaneous or mucosal contact with cannabis due to his reported clinical history and skin testing. Due to the inconsistences in strain identification by history and skin testing, he was advised to avoid all strains. An epinephrine autoinjector was prescribed in case he developed a more severe systemic reaction on repeat exposure. A telephone follow-up visit was conducted 6 months after the initial consultation. The patient endorsed that he was still smoking cannabis regularly but had not had any new significant reactions. He continued to use all above tested strains of cannabis in addition to a few new ones. He was still avoiding direct cutaneous and mucosal contact as much as possible.

## Discussion and conclusions

Despite the fact that all current strains of cannabis are believed to be descendants of two plant species, there is immense variability amongst commercially available strains. This patient had positive testing to three of five strains he was tested against, and he appeared to be more sensitized to Sativa dominant strains (Sweet Island Skunk, Blue Dream) as opposed to Indica dominant strains (Blue Moonshine). However, these results were inconsistent with the patient’s history as he believed that Blue Moonshine was the strain responsible for his prior severe local allergic reaction. We believe this is the first reported case of selective cannabis strain allergy based on patient history and skin prick testing.

There are currently only two allergens (Can s 3 and Can s 4) listed in the WHO/IUIS Allergen Nomenclature Sub-committee, and they were both identified in *C. sativa*. There are currently no known allergens of *Cannabis indica* listed in the database*.* It is estimated that only 72% of patients with a reported allergy to cannabis are sensitized to Can s 3 [10]. Published data is not currently available regarding the allergenicity of Can s 4. One study looking at genotyping of various cannabis strains showed that *C. sativa* and *C. indica* have distinguishable pools of genetic diversity and there is only a moderate correlation between the genetic structure of marijuana strains and their reported *C. sativa* and *C. indica* ancestry [11]. In addition, the immense amount of cross-breeding of different strains, and large number of strains currently commercially available, makes genetic analysis of cannabis plants difficult. With rising use of cannabis, the ability to accurately detect individuals with cannabis allergy on skin prick testing is important in order to reduce hypersensitivity reactions.

In the near future, cannabis extracts will likely be available for clinical use. As there are currently only two confirmed allergens from cannabis plants (both derived from *C. sativa*), this report also emphasizes the importance of further research into determining the specific allergens implicated in cannabis allergy. Having a larger number of identified allergens available will likely increase the specificity of testing. Accurate identification of cannabis allergens and incorporation into commercial extracts will undoubtedly be a difficult task given the complexity and variability of currently available commercial strains.

## Data Availability

Not applicable.
